# The significance of alternative transcripts for *Caenorhabditis elegans* transcription factor genes, based on expression pattern analysis

**DOI:** 10.1186/1471-2164-14-249

**Published:** 2013-04-15

**Authors:** Hannah L Craig, Julia Wirtz, Sophie Bamps, Colin T Dolphin, Ian A Hope

**Affiliations:** 1 School of Biology, Faculty of Biological Sciences, The University of Leeds, Leeds LS2 9JT, UK; 2 Institute of Pharmaceutical Science, King’s College London, 150 Stamford Street, London SE1 9NH, UK

## Abstract

**Background:**

Sequence-specific DNA-binding proteins, with their paramount importance in the regulation of expression of the genetic material, are encoded by approximately 5% of the genes in an animal’s genome. But it is unclear to what extent alternative transcripts from these genes may further increase the complexity of the transcription factor complement.

**Results:**

Of the 938 potential *C. elegans* transcription factor genes, 197 were annotated in WormBase as encoding at least two distinct isoforms. Evaluation of prior evidence identified, with different levels of confidence, 50 genes with alternative transcript starts, 23 with alternative transcript ends, 35 with alternative splicing and 34 with alternative transcripts generated by a combination of mechanisms, leaving 55 that were discounted. Expression patterns were determined for transcripts for a sample of 29 transcription factor genes, concentrating on those with alternative transcript starts for which the evidence was strongest. Seamless fosmid recombineering was used to generate reporter gene fusions with minimal modification to assay expression of specific transcripts while maintaining the broad genomic DNA context and alternative transcript production. Alternative transcription factor gene transcripts were typically expressed with identical or substantially overlapping distributions rather than in distinct domains.

**Conclusions:**

Increasingly sensitive sequencing technologies will reveal rare transcripts but many of these are clearly non-productive. The majority of the transcription factor gene alternative transcripts that are productive may represent tolerable noise rather than encoding functionally distinct isoforms.

## Background

Knowledge of the control of gene expression by complex regulatory networks acting at multiple levels is central to an understanding of metazoan development. Transcription factors, with the ability to distinguish between genes through their sequence-specific DNA-binding activity, have a key role at the primary level of gene expression, transcription. The large proportion of the genome devoted to encoding transcription factors increases as the size of the genome increases, highlighting their significance to biological complexity, and is around 5% for metazoans [1]. Alternative transcripts from these genes may increase the complexity of the transcription factor complement still further [2]. Increasingly sensitive sequencing technologies are revealing increasing numbers of alternative transcripts but it is unclear how much these add to transcription factor functionality. Transcription factor regulation of transcription factor genes is at the very heart of regulatory networks controlling expression of the genome. The complexity of interactions within transcription factor regulatory networks [3], further complicated by alternative isoforms, means that their comprehension depends on a more holistic view than the investigation of a single transcription factor can provide. The anatomical simplicity of the model system *Caenorhabditis elegans* provides a good subject for such systems scale analyses.

An extensive bioinformatics study, based on gene ontology and on DNA sequence predicted to encode known DNA-binding domains, identified 938 potential transcription factor genes in the *C. elegans* genome [4,5]. Many of these loci, however, are annotated as producing more than one transcription factor isoform and so significantly more than 938 transcription factors may potentially be encoded in the *C. elegans* genome. A gene can have alternative promoters, alternative transcription termination or undergo alternative splicing to encode discrete transcripts with distinct protein-coding regions. Approximately 15% of *C. elegans* transcription factor genes were identified as alternatively spliced [4]. This may be an under-estimate as the proportion of all *C. elegans* genes predicted or confirmed to encode alternative transcripts has increased from around 10% [6,7] to 25% [8] in recent years as the sensitivity of techniques has increased. The number of potential alternative transcripts for transcription factor genes is likely to increase still further, although how many of these transcripts actually encode functional distinct transcription factor isoforms or are simply noise in the system is not yet clear.

It may be anticipated that alternative transcription factor isoforms would be produced for distinct purposes. The different modes of transcript production could allow distinct expression patterns with consequent distinct distributions of the transcription factor isoforms encoded by a single gene, for distinct functions in different sets of cells. Alternatively, the different isoforms encoded by a single gene might have different functions in the same cells. Through interactions with different co-factors, mediated by alternative protein domains, or even through differences in the sequence recognition specificity of the DNA-binding domain, the different isoforms may regulate different sets of genes. In this case the different transcripts encoded by the transcription factor gene would show overlapping expression.

Gene expression patterns are most easily determined with single cell resolution, in terms of the largely invariant developmental cell lineage described for *C. elegans*, using reporter gene fusion technology. Historically this has involved the creation of reporter gene fusions with parts of the gene of interest within a plasmid context, as recently when specifically targeting alternative splicing in *C. elegans*[9]. However, genes with alternative transcripts are typically larger than genes without as they include alternative exons and associated regulatory elements. To maximize the chances of preserving the alternative modes of expression the whole of the gene and its genomic environs should be included, with minimal alteration, in any reporter gene fusion meant to tackle this subject. Seamless recombineering [10] of fosmids is an approach that addresses these issues.

Recombineering allows the precise insertion of a reporter gene into approximately 40 kb genomic DNA fragments cloned in fosmids. The compact genome of *C. elegans* means such fosmids typically contain the gene of interest along with several flanking genes and therefore the gene is present within the broader DNA context present endogenously. Specific exons can be tagged with the reporter to reveal the cells in which particular alternative transcripts can be found. Furthermore, such manipulated fosmids can be subjected to subsequent further recombineering steps to change single base pairs and thereby specifically eliminate contributions of certain transcripts to reporter expression patterns with minimal alteration [11,12].

*C. elegans* transcription factor genes thought to encode multiple transcription factor isoforms were selected for analysis. Prior evidence for the existence of alternative transcripts for these genes was assessed. Recombineering was then applied for each gene selected to create a range of corresponding reporter gene fusion arrangements. Reporter expression patterns were determined in *C. elegans* transformed with these fosmids to reveal if alternative transcripts tend to exhibit alternative spatio-temporal distributions in this species.

## Results and discussion

### Assessment of prior evidence for alternative transcription factor isoforms

Before any reporter gene fusions were generated we first invested some time in compiling a list of *C. elegans* transcription factor genes likely to encode multiple isoforms. Since the compendium of 934 *C. elegans* transcription factor genes used as the starting point was originally published [4] a few genes have been added or removed [5]. Of the 938 potential *C. elegans* transcription factor genes in the compendium at the time of our initial assessments, WormBase WS190 (see http://ws190.wormbase.org/) provided no evidence for the existence of alternative transcripts for 677. For 64 more genes, the alternative transcripts identified only differed in an untranslated region and therefore encoded the same protein. This left 197 *C. elegans* transcription factor genes annotated as encoding at least two distinct isoforms (Table 1). The evidence for and nature of the alternative transcripts encoding these distinct isoforms was evaluated (Additional file 1), with continuous re-assessment through to the most recent WormBase data freeze, WS230 (see http://legacy.wormbase.org/).

**Table 1:** Numbers of transcription factor genes encoding alternative isoforms

**Category**	**Number of transcription factor genes**
Total considered	938
No evidence for alternative transcripts	677
Alternative transcripts encode same protein	64
Alternative transcripts annotated to encode different isoforms	197
Alternative unique starting exon - strong evidence	21
Alternative unique starting exon - weak evidence	7
Nested alternative starts	22
Alternative terminal exon - strong evidence	0
Alternative terminal exon - weak evidence	23
Non-constitutive internal exon - strong evidence	0
Non-constitutive internal exon - weak evidence	5
Non-constitutive internal intron - strong evidence	3
Non-constitutive internal intron - weak evidence	3
Alternative splice site selection - strong evidence	4
Alternative splice site selection - weak evidence	20
Alternative transcripts by multiple mechanisms - strong evidence	9
Alternative transcripts by multiple mechanisms - weak evidence	25
Alternative transcript evidence considered invalid	55

Footnote: The names of each gene placed in each category above are provided in Additional file 1: Table S1.

**Figure 1:**
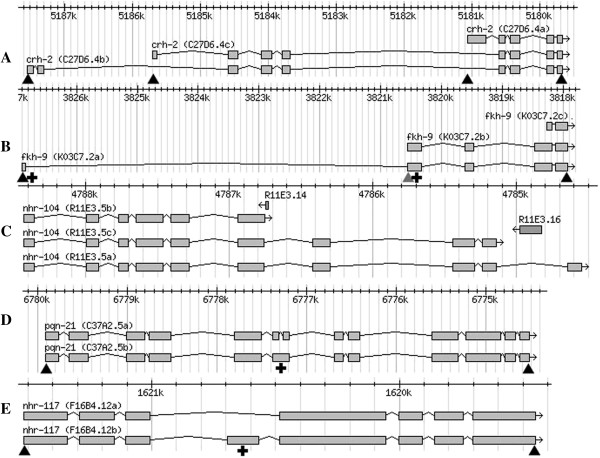
**Examples illustrating different modes of alternative transcript generation for *C. elegans *transcription factor genes. A**. Alternative promoters with unique starting exons for *crh-2*. **B**. Alternative promoters with nested transcripts for *fkh-9*. **C**. Alternative transcript ends for *nhr-104*. The non-coding RNA genes *R11E3.14* and *R11E3.16* are transcribed in the opposite direction. **D**. Inclusion or exclusion of an intron for *pqn-21*. **E**. Inclusion or exclusion of an exon for *nhr-117*. For each example, the molecular gene names are included in brackets after the genetic gene name, with the additional final letter (a/b/c) distinguishing the transcripts encoding distinct isoforms. The triangles indicate positions of *gfp* insertion used to tag expression for different transcripts. The grey triangle indicates that *gfp* was inserted with an extra nucleotide upstream to disrupt the translational reading frame, and therefore reporter expression, for other transcripts starting further upstream. The crosses indicate where translational reading frames of protein-coding regions were disrupted by the insertion of single base pairs to eliminate reporter expression arising from particular transcripts for fusion genes with *gfp* inserted at the end of the protein coding region. The scale bar in each panel is in base pairs along the respective chromosome.

The most common mechanism for generation of alternative transcripts amongst *C. elegans* transcription factor gene annotations was through different transcription start points, theoretically through the use of distinct promoters. This has been referred to as a “two-promoter system” [13] although a gene can have more than two. In the clearest examples within our transcription factor list, e.g. *crh-2* (Figure 1A), each alternative transcript starts with a different protein coding exon that is unique to that transcript and not found in any other transcript. For 21 transcription factor genes there was strong evidence for this gene structure being the sole mechanism for generation of alternative transcripts, with multiple independent ESTs (expressed sequence tags) identified for each transcript. Another 7 transcription factor genes were annotated as having alternative transcripts with unique starting exons, based on gene-directed RT-PCR amplification, but for which EST supporting evidence (WormBase) was either weak or absent. These genes, along with the genes with nested transcripts, were catalogued as *possibly* transcribed with alternative promoters. There were 22 genes for which annotation of alternative promoters was based solely on nested transcripts, where the start of the shorter transcripts lay within exons of the longest transcript, e.g. *fkh-9* (Figure 1B). One interpretation of nested transcripts is that an intron of the longer transcript contains an additional promoter that can initiate alternative transcription from a position within the immediate downstream, and otherwise internal, exon. Typically, however, only one of such a gene’s transcripts is well represented in the EST data and this leads to alternative interpretations. If a longer transcript is poorly represented, it may arise from spurious transcription initiating upstream of the gene and continuing, unarrested, through the transcription unit. If a shorter transcript is poorly represented, it may arise either biologically, from aberrant *trans*-splicing onto an internal splice acceptor or from a cleaved transcript, or technologically, from incomplete first strand cDNA synthesis. Such low abundance transcripts may well be detected with highly sensitive, gene-directed PCR approaches. This then raises the question of whether such transcripts, arising either from background noise in gene expression or from specific low-level promoters, are nevertheless functionally important. Of course low levels of transcript may reflect high levels of expression with considerable spatial or temporal restriction and consequent functional significance. Expression pattern analysis could distinguish examples of the latter.

In contrast to alternative transcriptional initiation, no convincing examples of transcription factor genes with variation in transcript end as the sole mechanism for generating alternative transcripts were found. The strongest examples of genes annotated as such in WormBase, 23 in number, proposed a single alternative exon, either internal or terminal, but typically based on a single EST. When the alternative exon is internal the theoretical open reading frame rapidly reaches a termination codon, well before the end of the annotated transcript, either within the alternative exon or because the translational reading frame is shifted from the correct reading frame in the immediate downstream exon. Nonsense-mediated decay (NMD) would be expected to degrade such transcripts [14]. More commonly there is a single alternative terminal exon that is simply an extension of an exon, internal to the gene, into what would otherwise be intron. Two foreshortened transcripts of this type are annotated for *nhr-104* (Figure 1C), one based on a single EST but the other based on several. Strikingly, however, for both foreshortened *nhr-104* transcripts the final exons are closely followed by non-coding RNA genes (*R11E3.14* and *R11E3.16*) that are transcribed from the opposite direction. It is tempting to speculate that colliding transcription complexes could lead to premature termination of *nhr-104* transcription and the ESTs found. Similar scenarios are seen in many other *C. elegans* genes. Another striking example of alternative transcript ends is provided by *nhr-88*. The alternative transcript termination for *nhr-88* is simply due to the presence of a transposable element that is sometimes included, leading to premature termination of the translational reading frame, and sometimes spliced out of the transcript. Again, to emphasize, the proteins encoded by such low abundance, aberrant, foreshortened transcripts may still be functionally important.

A number of *C. elegans* transcription factor genes are annotated with alternative transcripts generated solely by alternative splicing. Amongst these genes, the most distinct isoforms would arise through inclusion versus exclusion of entire internal introns or exons, e.g. *pqn-21* and *nhr-117* respectively (Figure 1D and E). The evidence for any transcription factor gene using a non-constitutive exon as the sole mechanism for creating an alternative transcript is weak, with just five examples and only one or two ESTs in support of the alternative transcript in each case. There are, however, six transcription factor genes where non-constitutive introns are annotated as the sole source of transcript variation and for three of these, *F23F12.9*, *zip-1* and *atfs-1*, the evidence is strong with many ESTs in support. Alternative splicing that gives more subtle isoform differences occurs where it is simply splice site selection that appears to vary. There are 24 transcription factor gene annotations with donor or acceptor splice site selection as the sole mechanism of transcript variation, but for almost all of these the alternative splice sites are just 3, 6, 9 or 12 nucleotides apart, thereby retaining the translational reading frame. Furthermore, for most of these, and for all three genes with alternative splice sites slightly further apart, there are only one or two ESTs to support the existence of the alternative transcript. There is good EST evidence, however, for alternative transcripts for *hmg-1.2*, *nhr-14*, *bed-1* and *Y65B4BR.5*, with just 3 or 6 nucleotide differences between the alternative splice sites for each. Although such small distinctions may lead to inclusion or omission of only one or two amino acids in the protein product, the structural and functional consequences could still be highly significant. Alternatively, these examples of splice site selection variation may still simply reflect tolerable noise in the expression of these genes. The fact that for all 24 examples the distinct transcripts differ by a number of whole codons, such that the translational reading frame is maintained, may only reflect fully translated transcripts not being substrates for NMD [14]; i.e. transcripts generated with alternative splice sites that do shift the translational reading frame would not be translated along their length, would be degraded by NMD and consequently would not have been detected.

For some *C. elegans* transcription factor gene annotations the derivation of alternative transcripts is more complex, involving combinations of the mechanisms above. The experimental evidence is strong for 9 such genes, although there are a further 25 for which the evidence is weaker. Strikingly, amongst the instances that are well supported, there are 7 genes with alternative exons and 4 with alternative transcript ends. As there are no well-supported instances of these mechanisms being the sole mechanisms for alternative transcript generation, perhaps such variations in *C. elegans* gene expression are interdependent or dependent upon alternative transcript initiation. Might selection of an alternative exon be dependent upon the transcript secondary/tertiary structure, with this in turn dependent upon the alternative transcript starts?

This left another 55 transcription factor genes for which the WormBase annotation as encoding distinct isoforms was discounted. Gene annotations with examples from each of the mechanisms for generating alternative isoforms were placed in this category. The generally low level of expression of transcription factor genes [15] does mean there is often little of the EST evidence needed to strongly support gene structures and makes it harder to distinguish biologically relevant transcription from background transcriptional noise. Therefore, the distinctions between a transcription factor gene likely encoding or possibly encoding or discounted from encoding alternative isoforms will not be sharp. Nevertheless, many genes were placed in the discounted category for good reason. For some the EST upon which an alternative transcript was based was most unlikely to be translated into a protein because of out-of-frame translation initiation codons in the proposed 5′UTR. For others, translation of the proposed transcript required use of a different translational reading frame from that encoding the transcription factor. Finally, for several genes the alternative annotated transcript simply corresponded to the primary unspliced transcript. But it remains possible that functionally significant isoforms may arise from genes in the discounted category, or even from those not yet even annotated as encoding distinct isoforms, either at low abundance or only under certain environmental conditions.

With the *C. elegans* transcription factor genes categorized in this way, various criteria were used to select specific genes with which to assay the significance of alternative isoforms through recombineered reporter gene fusions. Genes were avoided if: *i*. the genes were not annotated to encode or discounted from encoding distinct isoforms; *ii*. the genes had already been thoroughly investigated by others; *iii*. the genes were not contained within a fosmid clone in the available fosmid library; *iv*. the transcription factor function of the encoded product was uncertain; or *v*. the distinct isoforms arose from alternative splice site selection and differed by just a few amino acids. Given their numerical superiority amongst the transcription factor genes with strong support for alternative isoforms, particular attention was placed upon the genes with alternative promoters. Eighteen such genes were investigated, along with a few of the genes encoding isoforms generated by other mechanisms; 2 with non-constitutive exons, 3 with non-constitutive introns and 6 complex (Table 2). The gene models with their alternative transcripts are provided for all 29 assayed genes (Additional file 2). For all 121 recombineered reporter gene fusions, construction details and reporter expression patterns are described (Additional file 3). These details, along with fluorescence micrographs, are also provided in WormBase, and can be accessed using the URL http://www.wormbase.org/species/c_elegans/expr_pattern/Exprxxxx, where Exprxxxx refers to the WormBase expression pattern identification number (WBID). A few illustrative fluorescence micrographs are provided here for exemplar genes.

**Table 2:** Transcription factor genes investigated

**Molecular gene name**	**Genetic gene name**	**Alternative transcript annotation in WormBase**	**Alternative isoform encoding assessment**	**Comment on annotation**	DNA binding domain *
*C07G2.2*	*atf-7*	Alternative promoters	Well-supported		bZIP
*ZC376.7*	*atfs-1*	Intron +/−	Well-supported		bZIP
*C27D6.4*	*crh-2*	Alternative promoters	Well-supported		bZIP
*R13H8.1*	*daf-16*	Alternative promoters	Well-supported		WH - Fork, AT Hook
*F33H1.1*	*daf-19*	Complex; alternative promoters & exon +/−	Possibly valid	EST evidence for alternative promoters, single EST for alternative internal exon	WH - RFX
*F13G11.1*	*dmd-6*	Alternative promoters	Possibly valid	nested transcripts only	ZF - DM
*T22B7.1*	*egl-13*	Alternative promoters	Well-supported		HMG box
*F28B12.2*	*egl-44*	Complex; alternative promoters & splice site variation	Well-supported		TEA/ATTS
*F26D12.1*	*fkh-7*	Alternative promoters	Possibly valid	nested transcripts only	ZF - C2H2 - 1 finger, WH - Fork
*K03C7.2*	*fkh-9*	Alternative promoters	Possibly valid	nested transcripts only	WH - Fork
*W02C12.3*	*hlh-30*	Complex; alternative promoters, intron +/− & exon +/−	Well-supported		bHLH
*T24H10.7*	*jun-1*	Alternative promoters	Well-supported		bZIP
*F54H5.4*	*klf-3*	Alternative promoters	Possibly valid	RT-PCR primer derived alternative start	ZF - C2H2 - 3 fingers
*F16B4.12*	*nhr-117*	Exon +/−	Possibly valid	two ESTs for alternative internal exon	ZF - NHR
*C01H6.5*	*nhr-23*	Alternative promoters	Well-supported		ZF - NHR
*C45E5.6*	*nhr-46*	Alternative promoters	Well-supported		ZF - NHR
*T09A12.4*	*nhr-66*	Alternative promoters	Well-supported		ZF - NHR
*F26H11.2*	*nurf-1*	Complex; alternative promoters & transcript ends	Well-supported		AT Hook
*T28H11.4*	*pes-1*	Alternative promoters	Possibly valid	nested transcripts only	WH - Fork
*C37A2.5*	*pqn-21*	Intron +/−	Possibly valid	single EST for extra intron	ZF - C2H2 - 1 finger
*C47C12.3*	*ref-2*	Alternative promoters	Possibly valid	nested transcripts only	ZF - C2H2 - 3 fingers
*K08A8.2*	*sox-2*	Alternative promoters	Well-supported	nested transcripts only	HMG box
*C07A12.5*	*spr-3*	Intron +/−	Possibly valid	single EST for extra intron	ZF - C2H2 - 7 fingers
*ZK867.1*	*syd-9*	Complex; alternative promoters, exon +/− & splice site variation	Possibly valid	nested transcripts, single EST for extra exon & splice site variant	ZF - C2H2 - 4 fingers
*T28F12.2*	*unc-62*	Complex; alternative promoters & exons +/−	Well-supported		HD - TALE
*C30A5.7*	*unc-86*	Exon +/−	Possibly valid	single EST for alternative internal exon	HD - POU
*F14F3.1*	*vab-3*	Alternative promoters	Well-supported		HD - PRD
*F55H12.6*	*ztf-26*	Alternative promoters	Well-supported		ZF - C2H2 - 3 fingers
*F13H6.1*		Alternative promoters	Possibly valid	single EST for alternative unique exon	ZF - C2H2 - 3 fingers

Footnote: * Key to abbreviations: *bHLH* basic region helix loop helix, *bZIP* basic region leucine zipper, *HD* Homeodomain, *HMG* High mobility group, *TEA/ATTS* Transcriptional enhancer activator, *WH-Fork* Winged helix – Fork head, *WH-RFX* Winged helix – X-box binding regulatory factor, *ZF* Zinc finger, *ZF-DM* Zinc finger, Dsx and Mab-3-like, *ZF-NHR* Zinc finger, Nuclear hormone receptor.

### Alternative promoters with distinct starting exons

For each transcription factor gene examined, alternative promoters with distinct starting exons drove fosmid based reporter expression in only partly similar patterns, patterns neither all identical nor all discrete. The simple model of alternative promoters being present to allow expression of either functionally equivalent transcription factors in different cells or functionally distinct transcription factor isoforms in the same cells did not apply. Different promoters in a single transcription factor gene had different strengths, contributing to different degrees to the level of expression, and appeared to be under the influence of the same enhancers. It is also possible that the common components arise independently from the alternative promoters operating in isolation, through distinct sets of *cis*-acting regulatory elements. Some of the promoters in transcription factor genes drove expression only in subcomponents of the expression pattern driven by other promoters of the same gene. Often the expression from these transcription factor gene promoters was very broad, in multiple tissue types and throughout development.

The significance of distinct promoters for a transcription factor gene was most easily assessed when the promoters were identified from the presence of distinct starting exons for the alternative transcripts. Reporter genes were inserted immediately after the initiation codon in the starting exon of each transcript and also before the termination codon common to all transcripts (Figure 1A). Expression patterns of the former were expected to sum to the expression pattern of the latter.

All three alternative transcripts annotated for *vab-3*, i.e. vab-3a, vab-3b and vab-3c, have unique starting exons. When *gfp* was inserted immediately before the termination codon shared by all three transcripts, reporter expression was observed quite broadly, throughout development in the hypodermis, in body wall muscle in the anterior, in head and ventral cord neurons, in the intestine and in the distal tip cells (WBID Expr9742). A similar if not identical expression pattern, but weaker, was observed when *gfp* was inserted immediately after the initiation codon for transcript b (WBID Expr9731). While the common exons of the three *vab-3* transcripts lie in just over 2 kb of genomic DNA and the unique starts of transcripts b and c lie within the 1 kb upstream, the specific start of transcript a lies more than 10 kb away. Nevertheless, the promoter for transcript a appears to have a similar activity to the promoter for transcript b, but weaker again. When *gfp* was inserted after the initiation codon for transcript a, expression in head muscle and hypodermis were observed (WBID Expr9765). The absence of intestine and distal tip cell components may be due to the weakness of expression. Isolated promoter regions, as cloned in the Promoterome [16], typically drive much stronger reporter expression for transcription factors in comparison to insertion of the reporter into the whole gene [15]. The Promoterome fragment for *vab-3* transcript a [17] drove expression in the same components as observed with the other recombineered *vab-3* reporter gene fusions, including the intestine and distal tip cells (WBID Expr7504). The common expression pattern is particularly noteworthy given that the Promoterome fragment assayed is just 2 kb of DNA from upstream of the transcript a start, well away from the common exons shared by all three transcripts and the start of the other two transcripts. Meanwhile, the promoter for transcript c, located between the common exons and the start of the other transcripts, drove stronger although still weak reporter expression but with a much more restricted distribution, in just a few head nerve cells (WBID Expr9757).

For several other transcription factor genes examined, at least one of the promoters defined by a unique starting exon also appeared to drive expression in the same pattern as the gene in its entirety. For both *atf-7* and *nhr-46*, there are two promoters defined by unique starting exons, the most upstream of each looking dislocated from the rest of what are otherwise compact genes. Nevertheless, when *gfp* was inserted into either of the unique starting exons for *atf-7*, the same, strong, very broad specificity, reporter expression pattern, peaking in the L1 stage, was observed (WBIDs Expr9790/9808). For *nhr-46*, *gfp* inserted after the transcript a specific start codon, in the proximal of the two unique exons, gave the same expression pattern as when *gfp* was inserted before the termination codon common to all transcripts; again a broad distribution but peaking this time in the L2 stage (WBIDs Expr9816/9835).

For *ztf-26* and *egl-13*, *gfp* inserted after the initiation codon of the a transcripts, in the more distal unique exon, gave apparently the same reporter expression as when *gfp* was inserted before the shared termination codon of each gene. While the expression of *ztf-26* was broad, including nerve cells, hypodermis and muscle peaking in the L4 stage (WBID Expr9838), that for *egl-13* was more restricted, limited to a few nerve cells in the head and tail, and weakly in body wall muscle, but through all postembryonic stages (WBIDs Expr9735/9745). Again, the Promoterome fragment for *egl-13* transcript a [17] drove the same but much stronger reporter expression with detectable GFP production in vulval muscle as well as body wall muscle (WBID Expr7672). The recombineered reporter gene fusion to assay *egl-13* transcript d, however, gave weak but clear expression in the region of the developing gonad, probably the developing vulval muscle (WBID Expr9746). The impression given is that promoters for *egl-13* transcripts a and d both drive expression in the same cells, but with different strengths in different places, the very weak expression from promoter d being detectable in the developing vulva but not elsewhere, with the assay performed. For *ztf-26* the recombineered reporter gene fusion for the second promoter, that for transcript b, reveals a subtle distinction in promoter activity that may be more temporal rather than spatial, with GFP expression peaking in the L1 stage (WBID Expr9844).

Alternative promoter activity was confirmed for two genes, *F13H6.1* and *klf-3*, for which the EST evidence for the alternative promoter was considered weak. The unique starting exon of *F13H6.1a* was based on a single EST clone, yet *gfp* insertion after the initiation codon (WBID Expr 9727) gave the same strong reporter expression as insertion before the termination codon common to both *F13H6.1* transcripts (WBID Expr9843). Although not specifically assayed *F13H6.1b* would not appear to contribute any additional components to this gene’s expression pattern. Exon 1 of transcript a of *klf-3* (called *mua-1* in earlier versions of WormBase) starts just a few nucleotides before exon 2 of transcript b, after which the two transcripts are identical. The experimental evidence for the transcript a start is an RT-PCR derived ORFeome [18] clone generated using a 5′ PCR primer that included nucleotides corresponding to that transcript start (WormBase). Nevertheless, insertion of *gfp* after either initiation codon or before the termination codon yielded reporter expression and with closely related patterns (WBIDs Expr9810/9814). The absence of the intestinal component for the transcript a specific fusion was the only difference suggesting the transcript b promoter drives the full expression pattern for *klf-3*.

The *jun-1* gene has five promoters defined by unique starting exons and is probably expressed in all cells. The strong and broad expression driven by the *jun-1* transcript a Promoterome fragment [17] (WBID Expr7681) made it difficult to be certain that there were no cells lacking GFP, but the reporter was expressed more strongly in some tissues than others. All five promoters were assayed by recombineering-mediated insertion of *gfp* immediately after each of the unique initiation codons (WBIDs Expr9763/9764/9768/9780/9782). Once more, and for every promoter, the recombineered reporter gene fusions gave much weaker GFP expression than observed previously with the corresponding Promoterome construct. However, for fusions reporting on the different *jun-1* promoters, including that for transcript a, different components were emphasized with higher levels of expression and many components were shared in different combinations. The promoters for transcripts a and d appeared the strongest, while those for transcripts b and e were the weakest. Insertion of *gfp* upstream of the common termination codon gave the most widespread reporter expression for the recombineered fusions (WBID Expr9740). The impression given is that the broad expression of *jun-1* is generated in an overlapping piecemeal fashion, different isoforms lacking specific distributions of functional significance and possibly expressed to some level in all cells.

**Figure 2:**
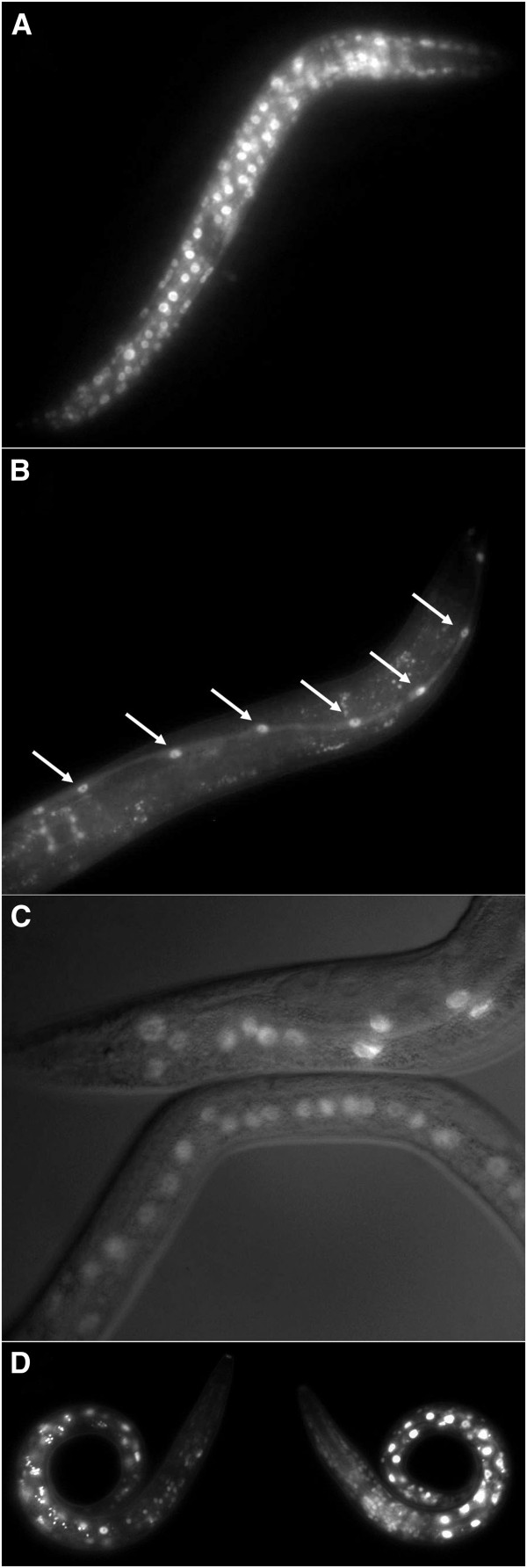
**Example fluorescence micrographs for expression of *crh-2::gfp *fusions. A**. An L2 larva of strain UL3706 with broad reporter expression for a fusion tagging transcript a. **B**. An L4 larva of strain UL3709 with reporter expression in the seam cells (nuclei arrowed) for a fusion tagging transcript b. **C**. An adult and an L4 larva of strain UL3809 with reporter expression in the intestinal nuclei for a fusion tagging transcript c. **D**. Two L1 larvae of strain UL3808 with broad reporter expression for a fusion tagging all three transcripts. The GFP is nuclear localized for each fusion. In **C** the fluorescence is superimposed upon the corresponding DIC micrograph. All images captured at 400x magnification.

Like *jun-1*, *crh-2* may also be expressed in all cells. However, while insertion of *gfp* immediately after the initiation codon unique to transcript a and before the stop codon shared by all three transcripts gave very broad expression (Figure 2A and D) (WBIDs Expr9788/9791), insertion of the reporter into the unique exons for each of the two more distally located promoters gave very specific expression. For transcript b and c, respectively, expression was observed only in seam cells in late larval stages (Figure 2B) (WBID Expr9789) and only in the intestine throughout development (Figure 2C) (WBID Expr9792). Two CRH-2 isoforms appear to be specifically expressed in certain cells while the third isoform is expressed more generally.

For two of the genes assayed, *nhr-23* and *nhr-66*, the two promoters defined by alternative starting exons did not appear to provide all the expression pattern components observed when the reporter was introduced before the common termination codon. While the hypodermal and neural expression was observed for the promoters of *nhr-23* transcripts c/d and a (WBIDs Expr9749/9781), the intestinal expression component of the terminal fusion was lacking (WBID Expr9736). Fusions for the promoters of *nhr-66* transcripts b and c (WBIDs Expr9750/9804) appeared to fail to drive the expression in the spermathecae or in the hypodermis and muscle throughout the body observed for other fusions (WBIDs Expr9766/9771). Weak reporter expression may again mean absence of expression components is simply due to the signal falling beneath background. The gene models for both of these genes do, however, have additional nested transcripts that could provide the missing components.

The *daf-16* gene is annotated as having 4 different unique starting exons. However, *daf-16* stretches over nearly 25 kb and was not contained fully in a single fosmid. Therefore, two overlapping fosmids were joined together by recombineering creating a fosmid with *daf-16* at the centre of a 65 kb insert. Although working with such a large clone was not straightforward, *gfp* was successfully inserted in two places. First, an insertion immediately before the stop codon tagged all annotated transcripts. Second, an insertion immediately after the transcript d start codon tagged transcripts d, f and h, the three longest transcripts, with clustered 5′ ends 12 kb upstream of the rest of the gene. Both of these reporter gene fusions and a previous conventional transcriptional reporter gene fusion to just the 2 kb region immediately upstream of the start of transcript a, only 3 kb from the 3′ end of the gene [17], yielded at least very similar GFP expression patterns (WBIDs Expr9815/9833/7644). Broad GFP expression in many tissues but most strongly in nerve cells was observed from early embryogenesis through to the adult.

### Alternative promoters with transcript starts located within exons of longer transcripts

Alternative promoters are also implied by gene model transcripts annotated to start within internal exons of longer transcripts (Figure 1B). Like transcripts with alternative unique first exons, such nested transcripts again share downstream exons and the termination codon. To assess the expression patterns of a nested transcript, *gfp* was inserted immediately after the proposed initiation codon. However, an extra base pair was simultaneously inserted upstream of the targeted initiation codon to shift the translational reading frame of, and eliminate reporter expression arising from, other transcripts within which the transcript of interest was nested. Alternatively or additionally, the recombineered fusion with *gfp* inserted before the termination codon was secondarily modified with single nucleotide changes in starting exons to successively eliminate reporter expression arising from alternative transcripts.

Typically, nested transcripts appeared expressed in the same pattern as the transcripts within which they were nested. For *ref-2*, insertion of *gfp* before the common termination codon or after the initiation codon for the longer transcript b or after the initiation codon for the nested transcript a but with translation from the upstream start disrupted, gave apparently identical reporter expression patterns (WBIDs Expr9812/9813/9829). This was quite a specific expression pattern consisting of the P blast cells and some of their immediate descendants, and a few nerve cells in the head and tail. For three other assayed genes with only nested alternative transcripts, *sox-2*, *dmd-6* and *pes-1*, the expression pattern appeared to be the same for all recombineered reporter gene fusions created for each (WBIDs Expr9798/9801/9841, Expr9817/9820/9821 and Expr9729/9753). Although not every possible reporter gene fusion arrangement was investigated in these cases, the absence of differences implies that nested transcripts do not provide additional expression pattern components beyond those provided by the transcripts within which they are nested. Nevertheless, the nested transcripts do appear productive. The reporter expression patterns of *sox-2* and *dmd-6* were broad, including several tissue types, while that for the forkhead gene *pes-1* was more developmentally restricted like *ref-2*.

**Figure 3:**
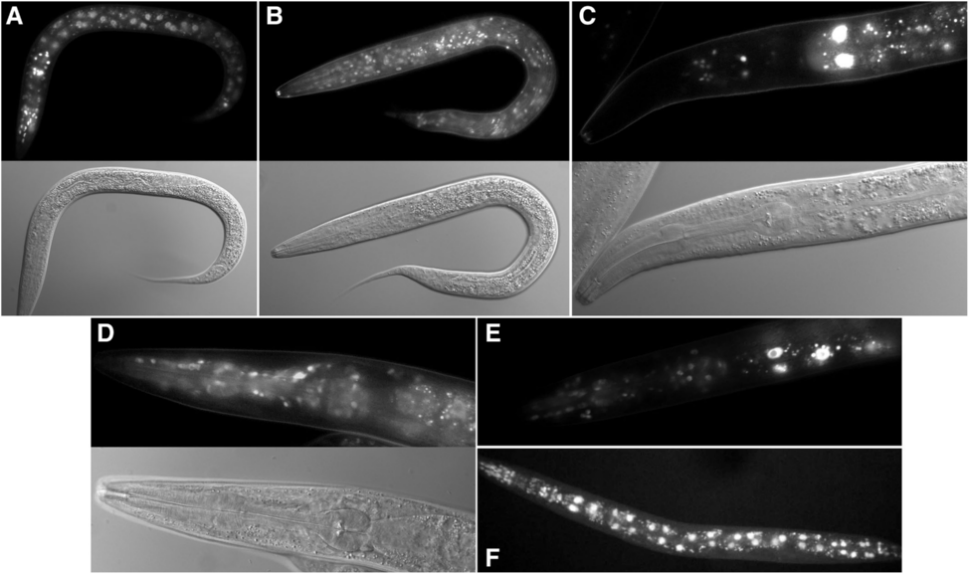
**Example fluorescence micrographs for expression of *fkh-9::gfp *fusions. A**. An L3 larva of strain UL3218 with *gfp* inserted before the *fkh-9* stop codon. **B**. An L3 larva of strain UL3146 with *gfp* inserted after the *fkh-9a* start codon. **C**. The head of an L4 larva of strain UL4076 with *gfp* inserted after the *fkh-9b* start codon. **D**. The head of an L4 larva of strain UL4089 with *gfp* inserted before the *fkh-9* stop codon but with exon 2 disrupted to eliminate expression of the reporter arising from *fkh-9a* and *fkh-9b*. **E**. The head of an adult of strain UL3998 with *gfp* inserted before the *fkh-9* stop codon but with exon 1 disrupted to eliminate expression of the reporter arising from *fkh-9a*. **F**. An adult of strain UL850 transformed with a plasmid-based reporter gene fusion that would reveal expression for transcript b, but not a or c. **A**-**D**. The corresponding DIC photomicrograph is provided beneath the fluorescence micrograph. **A**-**E**. captured at 400x, **F**. from [19] captured at 200x magnification.

Two other forkhead genes, *fkh-7* and *fkh-9*, were particularly thoroughly investigated. There are three annotated transcripts for *fkh-9*, all nested (Figure 1B). Most of the exons are contained within a 2.6 kb region but the first exon, only present in transcript a, is small (59 bp) and located 6.3 kb away, with an upstream intergenic region of just 316 bp. The smallest annotated *fkh-9* transcript, transcript c, starts towards the end of the penultimate fourth exon and would encode a protein of just 94 amino acids that does not include the DNA-binding forkhead domain. Transcript b, to which most ESTs correspond, starts with a trans-spliced leader added on to the second exon and has the appearance of the proper transcript for the gene. Despite this, insertion of *gfp* after the initiation codon of *fkh-9* transcript a or before the termination codon common to all annotated transcripts gave a similar broad expression pattern, with nuclear-localized GFP in many nerve cells, the intestine and the hypodermis, particularly in the head (Figure 3A and B) (WBID Expr9734/9738). When the reporter was inserted after the initiation codon of transcript b, with insertion of an extra base pair to eliminate translation from further upstream such as for transcript a, low levels of GFP were observed and only in the intestine and a few nerve cells in the head and tail (Figure 3C) (WBID Expr9830). The extra base pair insertion was placed between the directly juxtaposed transcript b initiation codon and the splice acceptor, and therefore may have interfered with this attempt to observe the expression for transcript b specifically. Indeed the alternative strategy, of eliminating reporter expression due to transcript a by disrupting exon 1 in the recombineered gene fusion with *gfp* inserted before the termination codon, appeared to leave the reporter expression pattern intact (Figure 3E) (WBID Expr9809). Now, clear expression in the nervous system, pharynx and intestine was also recorded as seen very strongly, previously, for a plasmid based reporter gene fusion reporting on *fkh-9b* and specifically not *fkh-9a* or *fkh-9c*[19] (Figure 3E) (WBID Expr2337). In addition to reporting on transcript b, however, this recombineered reporter expression could include contributions corresponding to transcript c and possibly other un-annotated transcripts. This scenario was confirmed from observations of the reporter expression arising when contributions due to both transcript a and transcript b were eliminated by disrupting exon 2 in the recombineered *fkh-9* fusion with *gfp* inserted before the termination codon (WBID Expr9834). Again, GFP was observed in nerve cells in the head and tail, with some expression in head hypodermis and intestine (Figure 3D). Nevertheless, the impression given is that *fkh-9* transcript b is the functionally important transcript but the distribution of background transcript noise, arising from either cryptic promoter activity (*fkh-9a*) or aberrant *trans*-splicing (*fkh-9c*), is influenced by transcript b regulatory elements.

The gene model for *fkh-7* appears very similar to that for *fkh-9*. Most *fkh-7* ESTs correspond to transcript b starting from exon 2. There is a small exon 1 far upstream starting transcript a and a small transcript c from the end of the gene. (A single-exon transcript, transcript d, is also annotated for the very end of *fkh-7* but with a different translational reading frame altogether and was therefore ignored.) When *gfp* was inserted before the termination codon common to transcripts a, b and c, reporter expression was seen from embryo to adult, and in nerve cells, hypodermis, pharynx, muscle, spermathecae, vulva and intestine (WBID Expr9732); again very broad expression as for *fkh-9*. Levels of the nuclear-localized GFP varied between tissues with the weaker components only seen in the transgenic strains with the strongest expression. Reporter expression was observed but at a lower level when *gfp* was inserted after the initiation codon of transcript a (WBID Expr9733), or after the initiation codon of transcript b with insertion of an extra base pair to eliminate translation from further upstream such as for transcript a (WBID Expr9822). Nevertheless, the GFP distributions arising ostensibly from the promoters specific for transcripts a or b appeared the same as when all transcripts were terminally tagged. When exon 5 was disrupted in the terminally tagged reporter fusion, to block expression arising from transcripts a and b, broad expression of GFP continued to be observed, but with the nuclear localization lost (WBID Expr9831). The only remaining annotated transcript, transcript c, would encode a protein lacking the DNA-binding forkhead domain, consistent with the loss of nuclear-localization. However, when expression due to transcripts a and b was disrupted with a two base pair deletion in exon 2, in the terminally tagged construction, broad nuclear-localized reporter expression was retained (WBID Expr9773). These observations suggest an alternative promoter can drive production of another un-annotated transcript for this gene, including more exons than found in transcript c, at least if expression from upstream promoters is perturbed. But again the different isoforms arising from the nested transcripts of *fkh-7* appear to be expressed in the same cells and the alternative promoters do not exist to confer expression in different locations.

Additional nested transcripts are identified in WormBase models for some of the genes considered in the preceding section, with alternative promoters identified from unique starting exons. Although not specifically assayed, the similarity of expression patterns for reporter gene fusions that were recombineered suggests that the nested transcript c for both *nhr-46* and *ztf-26* would not contribute any novel expression pattern components. The expression patterns for nested transcripts for three other genes of this type, *egl-13*, *nhr-23* and *nhr-66*, were specifically investigated. Again *gfp* was inserted immediately after the proposed initiation codon by recombineering with simultaneous insertion of a single extra base pair to eliminate translation arising from further upstream. From this approach, the *egl-13* transcript c appears to be expressed in the same pattern as transcript a within which it is nested (WBIDs Expr9800/9745). For *nhr-23*, *gfp* inserted into the unique starting exons of transcripts a and c/d failed to yield the clear intestinal component observed when *gfp* was inserted into the common final exon (WBIDs 9736/9749/9781). An assay aimed specifically at transcript b/f, nested within transcript a, did yield intestinal GFP, but inconsistently (WBID Expr9760). We considered the evidence for annotated *nhr-23* transcript e, nested within all the other transcripts, to be very weak so this transcript was not specifically assayed but presumably there is another transcript for *nhr-23* that is responsible for the intestinal component and this could be transcript e. However, for *nhr-66* the specific assay of transcript a yielded reporter expression (WBID Expr9771) very similar to that for *gfp* insertion in the shared terminal exon (WBID Expr9766) and much stronger than the extremely faint expression observed for transcript c (WBID Expr9804) in which transcript a is nested. EST support is also much stronger for transcript a than transcript c suggesting that the nested transcript a is actually the primary transcript.

The nested transcripts of *egl-44* were investigated, although the alternative splice site selection also in the annotation for this gene was not. Insertion of *gfp,* either before the termination codon common to all transcripts or after the start codon of the longest transcript (transcript a), yielded broad nuclear-localized expression (WBID Expr9761/9769). In contrast, insertion of the reporter immediately after the start codon for transcripts starting with the second exon, transcripts b/c, yielded very weak expression of much more limited distribution and lacking sub-cellular localization (WBID Expr9752). The extra base pair to disrupt reporter expression arising from translation from further upstream, had to be inserted immediately between the splice acceptor and the initiation codon and, therefore, could have perturbed expression for transcripts b/c more dramatically, as for *fkh-9b*. Nevertheless, nested transcripts for *egl-44* do not appear to add significantly to the expression of this gene.

### Optional internal introns and exons

While the bioinformatic analysis yielded few likely examples of alternative transcription factor isoforms being derived solely from alternative splicing, a sample of these were selected for investigation. The significance of alternative transcripts derived from alternative splicing could be tested by disrupting reporter expression that is specifically dependent upon translation across the optional protein-coding region. The reporter gene was inserted by recombineering at the start and end of the gene to observe expression due to all transcripts. Subsequently, single base pairs could then be inserted by recombineering into the optional exon or optional intron within the recombineered reporter fusion with *gfp* inserted before the termination codon. Such minimal manipulation should shift the translational reading frame and disrupt reporter expression arising from inclusion of that optional region in the transcript. Expression resulting from transcripts with the optional exon skipped or the optional intron spliced out should remain. Comparison of the remaining reporter expression to the expression observed for all transcripts could reveal the significance of the alternative splicing.

Three genes with potentially non-constitutive introns, *atfs-1*, *spr-3* and *pqn-21*, were selected. While *gfp* inserted immediately before the stop codon for *spr-3* only drove infrequent nuclear-localized reporter expression in individual embryonic cells (WBID Expr9762), the same type of fusion for *atfs-1* gave reproducible and broad non-nuclear-localized GFP (WBID Expr9743). A lack of reporter expression for conventional full-length *spr-3* fusions was also observed previously [20]. The *atfs-1* result is consistent with the similar expression pattern described for a conventional reporter gene fusion and this transcription factor only becoming nuclear-localized in response to stress [21]. However, no reporter expression was observed when *gfp* was inserted immediately after the start codon of *atfs-1* and *spr-3* (WBID Expr9728/9770). As a consequence, the significance of the alternative transcripts for these two genes was not explored further.

**Figure 4:**
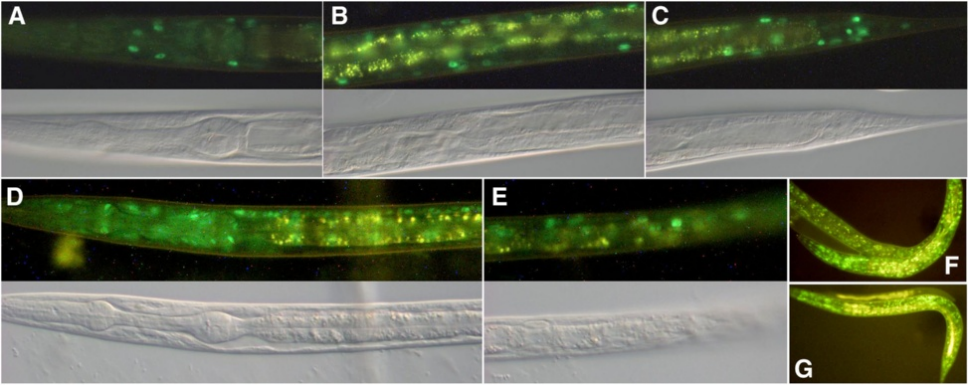
**Example fluorescence micrographs for expression of *pqn-21::gfp *fusions. A**-**C**. The head, mid-section and tail, respectively, of an L4 larva from strain UL3453 with *gfp* inserted after the *pqn-21* start codon. **D**, **E**. The head and tail, respectively, of an L2 larva from strain UL3412 with *gfp* inserted before the *pqn-21* stop codon. **F**, **G**. Adults from strain UL1131 with a plasmid based reporter gene fusion from [22] with *pqn-21* tagged with *gfp* at the 5^th^ exon. **A**-**E** The corresponding DIC photomicrograph is provided beneath the fluorescence micrograph. **A**-**C** captured at 200x, **D**, **E** captured at 400x, **F**, **G** captured at 100x magnification.

For *pqn-21* there was no fosmid available with the gene located centrally on the insert. The whole *pqn-21* protein-coding region is just included in fosmid WRM0637bF05, but with only 27 bp of downstream genomic DNA. In contrast, fosmid WRM0622dE07 extends a long way downstream but only contains 1 kb into the next upstream gene beyond the 1 kb intergenic region. Curiously, when *gfp* was inserted immediately after the *pqn-21* start codon in these two fosmids, reporter expression was only observed for the WRM0622dE07-derived reporter fusion suggesting DNA downstream of the WRM0637bF05 end point is important for *pqn-21* expression in full-length reporter gene fusions. With *gfp* inserted immediately before the termination codon or after the start codon of *pqn-21* in WRM0622dE07, nuclear-localized GFP was observed in apparently all somatic cells (Figure 4A-E) (WBID Expr9751/9754). The same result was obtained with a more conventional, plasmid-based, reporter gene fusion, with *gfp* tagged onto the first half of the gene, although the fluorescence signal was stronger [22] (Figure 4F and G) (WBID Expr2075). Insertion of a single base pair into the middle of the annotated optional intron (number 6 of 12 in transcript a) of the 3′ *pqn-21::gfp* fosmid-based fusion abolished detectable reporter expression (WBID Expr9784) suggesting that only the transcript skipping the splicing out of the annotated optional intron is productive. The implication is that there is no alternative transcript for *pqn-21* and annotated intron 6 is not really an intron.

**Figure 5:**
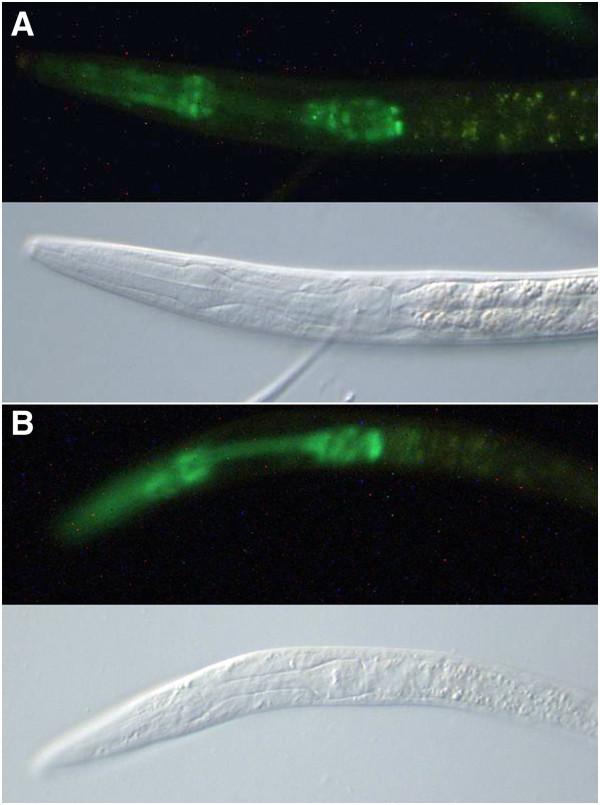
**Example fluorescence micrographs for expression of *nhr-117::gfp *fusions. A**. The head of an L1 larva of strain UL3468, with *gfp* inserted before the *nhr-117* stop codon. **B**. The head of an L1 larva of strain UL3209, with *gfp* inserted after the *nhr-117* start codon. The corresponding DIC photomicrograph is provided beneath the fluorescence micrograph and images were captured at 400x magnification.

Two genes with potentially optional exons as the sole means of generating alternative isoforms were investigated, *unc-86* and *nhr-117*. Nuclear-localized neuronal expression was observed when *gfp* was inserted into a fosmid by recombineering immediately upstream of the *unc-86* termination codon (WBID Expr9807), consistent with expression patterns described previously [23]. However, no expression was observed when the *gfp* was inserted immediately after the annotated start codon (WBID Expr9795) and therefore this gene was not investigated further. For *nhr-117*, *gfp* insertions at the end or start of the protein-coding region yielded the same reporter expression pattern, including the pharynx (Figure 5), intestine and cells in the rectal region (WBIDs Expr9737/9756). No reporter expression was observed when the annotated optional exon was disrupted by insertion of a single base pair into the 3′ *gfp* fusion (WBID Expr9805)*.* This suggests that the exon annotated as optional is actually required for productive expression of *nhr-117*.

Few examples of transcription factor isoforms being generated solely by alternative splicing were apparent from the bioinformatic analysis and the evidence therein was quite weak. Experimental investigation of five such genes failed to yield any evidence in support of such mechanisms being in operation for these genes.

### Alternative transcripts generated by multiple mechanisms

In contrast, there were 8 transcription factor genes for which there was good prior evidence for alternative splicing occurring in combination with the use of alternative promoters, and a further 19 for which the evidence for such was weaker. Expression patterns of 3 of the former and 2 of the latter genes were investigated. A few further genes appeared to combine alternative splicing with alternative termination but these were not examined experimentally.

**Figure 6:**
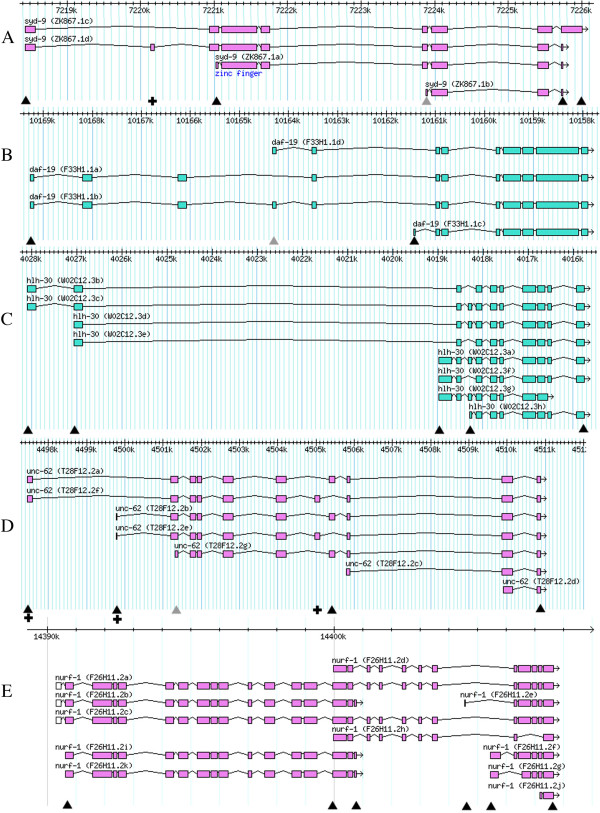
**Gene models for assayed genes with multiple mechanisms of alternative transcript generation. A**. *syd-9*. **B**. *daf-19*. **C**. *hlh-30*. **D**. *unc-62*. **E**. *nurf-1*. The molecular gene names are included in brackets after the genetic gene name, with the additional final letter (a/b/c etc.) distinguishing the transcripts encoding distinct isoforms. The triangles indicate positions of *gfp* insertion used to tag expression for different transcripts. The grey triangle indicates that *gfp* was inserted with an extra nucleotide upstream to disrupt the translational reading frame, and therefore reporter expression, for other transcripts starting further upstream. The crosses indicate where translational reading frames of protein-coding regions were disrupted by the insertion of single base pairs to eliminate reporter expression arising from particular transcripts for fusion genes with *gfp* inserted at the end of the protein coding region. For *nurf-1*, the first exons (white) of transcripts a, b and c were included in WS190, but not WS230. The scale bar in each panel is in base pairs along the respective chromosome.

The *syd-9* gene model (Figure 6A) has three alternative nested transcription starts and an alternative donor for the final intron, with the second exon only included infrequently in the longest transcript, according to the EST data. Reporter expression was observed in head and tail nerve cells when *gfp* was inserted immediately after the most upstream start codon, that for transcripts c/d (WBID Expr9799). In contrast, no reporter expression was observed when *gfp* was inserted after either of the alternative downstream start codons, for transcripts a and b, (WBIDs Expr9748/9806) suggesting that only the first transcription start contributes to expression of *syd-9*. Assay of the downstream start of transcript b involved incorporation of an extra base pair to prevent reporter expression from transcripts starting further upstream. This was unnecessary for transcript a, as the annotated first exon is in a different translational reading frame. The alternative splicing of the final intron means the gene model also proposes two alternative translational reading frames for the final exon. Reporter expression was observed when *gfp* was inserted immediately upstream of, and in frame with, the furthest downstream stop codon, that for transcript c (WBID Expr9794), but not with the other stop codon, for transcripts a/b/d (WBID Expr9758). These results again suggest that only the longer open reading frame is functional. Specific disruption of the translational reading frame of the optional second exon that appears in transcript d but not c, by insertion of a single base pair into the 3′ reporter fusion, had no apparent effect on GFP expression consistent with any inclusion of this exon making a minor contribution to *syd-9* expression (WBID Expr9803). This reporter expression analysis therefore only provides support for *syd-9* transcript c.

The gene model for *daf-19* also proposes three alternative transcriptional starts (Figure 6B). The shortest transcript (transcript c encoding DAF-19M) arises from the most downstream promoter and begins with a unique exon. The transcript from the middle promoter (transcript d encoding DAF-19C), however, starts in an exon designated as optional for the longest transcripts (transcript b with the exon, transcript a without) and is therefore nested. GFP expression was observed upon insertion of the reporter after each of the three alternative start codons (WBID Expr9747/9767/9796). The fusion to assay the nested transcript, transcript d, included a frame-shifting base pair insertion before the initiation codon to shift the reading frame and eliminate contributions to reporter expression from the most upstream promoter. Activity was therefore revealed for all three promoters, although the reporter expression for the nested transcript’s promoter was weak. The reporter expression patterns arising from the three promoters also appeared nested in that, in hermaphrodites, the most upstream promoter drove broad expression in neural and, weakly, in non-neural cells, the middle promoter drove expression in only neural cells but in the head and tail, while the most downstream promoter drove only neural expression and only in the head. Any subtle distinctions in these expression patterns within the neural system corresponding to the distinct *daf-19* transcript distributions described by others [24,25] would have needed more thorough analysis. The previously described male-specific head neuron expression for transcript c [25], the *daf-19* transcript labelled simply as male-specific in WormBase, was observed with the recombineered fusion specific for *F33H1.1c* (WBID Expr9747). However, strong male specific neural expression was also seen with fusions recombineered specifically for *F33H1.1a/b*, but in the tail (WBID Expr9767). Nevertheless, the distinct distributions of isoforms revealed previously by immuno-histochemistry using antibodies specific to the DAF-19 N and C-terminal regions [24] could be entirely due to differences in expression of the non-nested transcripts.

There are four transcript starts annotated for *hlh-30* (Figure 6C). One, for transcripts d/e, is nested from the second exon of the longest transcripts, transcripts b/c. Transcripts a/f/g start with an exon in the long intron of, and not included in, those four transcripts starting further upstream. Even further downstream in the gene model, there is an optional exon, included in transcripts a/c/d/g in which another nested transcript, transcript h, also begins. Finally, the second to last intron is optional, creating an alternative earlier translation termination when this splicing event is skipped, in transcript g. The annotation proposes 8 transcripts in total, as not every permutation is included.

When *gfp* was inserted before the final stop codon of *hlh-30* or after the start codons of the non-nested transcripts, i.e. transcripts b/c and a/f/g, the same broad, strong, nuclear-localised reporter fluorescence was observed in many tissues and from early embryogenesis onwards (WBID Expr9739/9774/9785). When the reporter was inserted after the initiation codon of transcripts d/e, the nested transcripts starting from the second exon, GFP was again expressed, and with a broad distribution, but was no longer nuclear-localized (WBID Expr9775). However, this fusion would also have tagged expression from transcripts b/c starting further upstream. Therefore, the non-nuclear-localization may arise from disruption of functionality of HLH-30b/c::GFP fusion proteins and, as the cellular distribution is indistinguishable, this expression does not provide support for transcripts d/e. An attempt to test if the alternative splicing was responsible for distinct distributions of HLH-30 isoforms was equivocal; insertion of *gfp* into the optional exon yielded no reporter expression (WBID Expr9776), suggesting that this exon is not included in functional transcripts, despite strong EST evidence of frequent inclusion of this exon. Nevertheless, the two distinct *hlh-30* promoters clearly drove strong expression of two HLH-30 isoforms in apparently the same cells throughout the soma.

There is good EST evidence for two alternative starting exons and two mutually exclusive internal seventh exons for *unc-62* (Figure 6D). The EST evidence in support of the three further nested transcripts in the gene model, each with their own transcription start points, is weaker. There were no fosmids available with the 13.5 kb gene centrally located with which to examine *unc-62* expression. Therefore the reporter was inserted by recombineering into two different fosmids, one extending further upstream of *unc-62*, the other further downstream, but both including at least 2 kb either side of the protein coding region. Both fosmids appeared to contain all essential *cis*-acting regulatory elements controlling *unc-62* expression as equivalent constructions in the two fosmids gave apparently the same reporter expression patterns. Insertion of the reporter immediately upstream of the termination codon common to all transcripts yielded nuclear-localized GFP in multiple discrete components including nerve cells, muscle cells, the vulva, the hypodermal seam cells and the intestine, and from embryogenesis to the adult (WBID Expr9786/9837). However, the relative strength of signal in the different expression components was noted to vary between different independent transgenic lines created with any particular fosmid-based reporter gene fusion. A possible interpretation is that *unc-62* is subject to complex self-regulation such that reporter fusion expression is sensitive to subtle variations in configuration of a few of the transgene copies present in the tandem extra-chromosomal arrays generated during *C. elegans* transformation. To examine expression arising specifically from the different transcripts the *gfp* reporter was inserted into one of the alternative first or seventh exons, or these non-constitutive exons were specifically disrupted by insertion of a single extra base pair in the 3′ reporter gene fusion fosmid constructions. All such fusions drove *gfp* expression in multiple locations suggesting that the different *unc-62* transcripts are not responsible for discrete expression pattern components and all are expressed in many locations (WBID Expr9777/9778/9787/9793/9811/9818/9819/9825/9839/9845). Indeed reporter expression was still observed in multiple components when both unique first exons were disrupted by recombineering of the 3′ reporter fusion (WBID Expr9832/9797), so these are not the only functional transcriptional starts. One other transcript, nested transcript g, was shown to be functional as when *gfp* was inserted immediately after the start codon, with a single base pair insertion to disrupt any translation arising from further upstream, multiple reporter expression components were again observed (WBID Expr9840). There appear to be many alternative *unc-62* transcripts each expressed in multiple components that are substantially, if not completely, overlapping.

The *nurf-1* gene model annotation (Figure 6E) has been modified repeatedly within WormBase (with some consequent confusion in transcript names) and has a particularly complex structure with 11 annotated transcripts, multiple promoters and two points of transcript termination. The 17kb *nurf-1* protein coding region was not contained in any single fosmid and so, as with *daf-16*, two fosmids were joined together by recombineering to reconstitute the whole unit before inserting the reporter. In addition to transcripts running through the entire gene model, there are transcripts for both halves of the gene that overlap in the two central exons. Insertion of *gfp* after the most upstream *nurf-1* start codon (in WS230), for transcripts a/b/c/i/k, yielded very broad nuclear-localized fluorescence (WBID Expr9824). This expression pattern represents expression arising for the transcripts from just the 5′ half of the gene plus the transcripts running the entire length of the gene. An essentially equivalent pattern of reporter expression was observed with *gfp* inserted at the end of the central alternative terminal exon (WBID Expr9826), which only tags transcripts b/i/k from the 5′ half of the gene, suggesting that transcripts running the whole length of the gene add little to the gene’s expression. Furthermore, insertion of *gfp* after the second annotated start codon, which initiates translation for transcripts d/h from across the 3′ half of the gene, also gave the same expression pattern (WBID Expr9827). As this construction would report on expression of transcripts from across just the 5′ as well as just the 3′ gene halves and the full-length transcripts, this observation suggests that the transcripts across the whole 3′ half of the gene also contribute little to the gene’s expression. There are a series of alternative transcription starts annotated close to the 3′ end of the gene, for transcripts e, f/g and j. Insertion of *gfp* after the initiation codon in the exon unique to transcript e yielded no detectable reporter expression (WBID Expr9828). Similarly, no expression was observed upon insertion of *gfp* immediately before the stop codon of transcript h, with the last exon in a different reading frame from all other transcripts for the 3′ half of the gene due to a proposed alternative splice. Transcripts e and h may not be functionally significant. Insertion of *gfp* after the initiation codon in the exon unique to transcripts f/g, however, yielded a strong signal that, in contrast to the other positive results for *nurf-1*, was not nuclear-localized, with a slightly more restricted tissue distribution (WBID Expr9842). The implication is that *nurf-1* primarily encodes two gene products, one corresponding to the 5′ half of the gene, which is nuclear-localized, and one to only the last 5 exons, which is not nuclear-localized, but occasional failure to terminate transcription centrally leads to rare transcripts across the entire gene. This interpretation is consistent with the distribution and abundance of *nurf-1* ESTs (WormBase). Nevertheless, the conservation of the entire *nurf-1* transcription unit organization in other animals suggests that the rare full-length transcripts are functionally significant, even if not very abundant.

Although the majority of GFP fusions made in this study gave detectable expression *in vivo*, neither of those tagging an alternative translational reading frame did. In both cases assayed, for *syd-9* and *nurf-1*, the alternative reading frame runs through the final exon, is based on a single EST, and could simply reflect a rare splicing error. Functional alternative translational reading frames are seen frequently in viral genomes, with severe size constraints, but rarely in animal genomes where there is much less pressure to use the genome in an efficient manner.

## Conclusions

The significance of alternative transcripts for the expression of *C. elegans* transcription factor genes was explored. Expression patterns were determined *in vivo* for 121 *gfp* reporter fusions, constructed in fosmids by recombineering to maintain the broad genomic DNA context, for 29 genes. We began with a thorough assessment of the gene models as annotated in WormBase. A *gfp* reporter was inserted seamlessly at specific points in each gene, with or without subsequent minimal manipulations, so as to assess the contributions of different transcripts to the complete expression pattern of each gene.

The alternative transcripts of *C. elegans* transcription factor genes encoding multiple isoforms differ most frequently in their starting points, their 5′ ends. Transcripts with distinct first exons presumably arise from distinct promoters. Strikingly almost all such genes yielded either a very broad or constitutive reporter gene fusion expression pattern, in marked contrast to the spatially and/or temporally restricted expression patterns seen more frequently in a prior, unfocussed screen of *C. elegans* transcription factor gene expression patterns [17]. Only *egl-13*, amongst the 17 examples examined, yielded a more restricted expression pattern but this still included nerve cells, body wall muscle cells and vulval muscle cells, through postembryonic development. The full broad gene expression pattern of each gene appeared driven by at least one of the promoters in every example, with other promoters driving expression in sub-components. There are hints from close observation of the reporter expression that this may actually reflect each promoter driving expression in all of the different components for a particular gene but at markedly different levels. Such a mode of expression could arise from the promoters being quite general, but regulated by multiple enhancer elements for different expression components distributed across the gene, their influence on each promoter varying, perhaps with proximity.

The other way in which transcripts for a gene can differ at their 5′ ends is when the transcripts are nested, with the starts of shorter transcripts lying within internal exons of longer transcripts. Again, five of the seven genes examined which had purely nested alternative transcripts yielded broad expression. It may have been anticipated that often such nested transcript annotations would simply reflect artefactual ESTs arising from truncated first strand cDNA synthesis with no biological relevance. However, each assay specific for a nested transcript, with elimination of expression arising from further upstream, yielded reporter expression. This suggests that typically the nested transcripts do indeed contribute to the expression of a gene and could have their own promoters. Nevertheless, the expression patterns observed for the nested transcripts typically appeared the same as for the transcripts within which they were nested. The single exception to this was *nhr-23* although the additional intestinal component observed with the reporter fusion for the nested transcript was only seen infrequently. In general, therefore, the nested transcripts may arise simply from transcription starting from the shared upstream promoter, with trans-splicing onto an internal, downstream splice acceptor to generate the shorter transcript(s). Such nested transcripts would not provide differential expression of transcription factor isoforms.

Dramatically distinct transcription factor isoforms would be generated if alternative transcripts arose from inclusion versus exclusion of optional introns or exons. However, examination of all our reporter expression patterns failed to provide evidence to support such an arrangement as the sole mechanism operating for a transcription factor gene. While alternative splicing of *C. elegans* genes has received considerable attention (reviewed in [26]) this mode of generating alternative isoforms appears of less importance for transcription factor genes and indeed few *C. elegans* transcription factor genes are annotated with such an organization. Furthermore, for 3 of the 5 genes specifically investigated here in this regard, insertion of the reporter after the annotated initiation codon failed to yield reporter expression, a result not seen for any of the other genes examined. The concerns over the gene models that such results raise was vindicated, at least for *unc-86*, with the subsequent revision of this gene’s start in WormBase revealing that the reporter had actually been inserted upstream, outside of the real protein coding region. But this only serves to emphasize the lack of certainty in gene structures that exists for genes, like those encoding transcription factors, with low levels of expression and consequent poor EST evidence. The gene models have been revised for 10 of the 29 assayed genes in the four years between WS190 and WS230. In addition, reporter expression observed for *pqn-21* and *nhr-117*, the other 2 genes of the 5 examined here, yielded no support for alternatively spliced transcripts. In both cases, the coding region annotated as optional appeared to be used constitutively, whether this is an exon (*nhr-117*) that is spliced into, or an “intron” (*pqn-21*) that is not spliced out of, the final transcript.

Reporter analysis did show alternative exons were utilised for *unc-62*, but in combination with other modes of alternative transcript generation and, uniquely, the alternative internal exons were mutually exclusive rather than optional. Further complexity in *unc-62* expression, beyond generation of the four previously characterized transcripts [27], is implicated: first, from the variability of expression pattern between different transgenic lines created with the same reporter gene fusion; second, from the continuation of reporter expression even after the coding regions of both of the mutually exclusive alternative first exons were disrupted. The latter expression could have arisen from one or more of the nested transcripts, one of which was confirmed to be functional. The promoter driving expression of the nested transcript could lie upstream of any of the first three exons, the uncertainty introduced by the complications of *trans*-splicing. Despite the range of transcripts encoded, *unc-62* expression was broad with multiple components and the components attributed to different transcripts appeared substantially overlapping if not identical. It was impossible to associate specific transcripts with specific components suggesting whatever the importance of having alternative transcripts, it is not to allow different UNC-62 isoforms to be expressed in distinct sets of cells.

Similarly, none of the other genes, with alternative transcripts generated by multiple mechanisms that were examined, yielded evidence for non-overlapping expression patterns for different isoforms. For *daf-19*, the expression patterns for the downstream promoters and nested transcript appeared to overlap substantially with the broad expression domain of the upstream promoter. For *hlh-30*, both distinct promoters gave the same broad expression, with the nested transcripts encoding fusion proteins that were no longer nuclear-localized. The complexity of the *syd-9* annotation may be misleading in that only one transcript appeared to yield reporter expression. Even for *nurf-1*, with its clearly distinct yet linked halves, expression is very broad, at least for the first half of the gene.

The general impression gained from this extensive analysis of a significant sample of *C. elegans* transcription factor genes is that structural qualities of this class of proteins, combined with the broad expression of certain members, simply allows alternative gene transcripts to be tolerated. It has been noted previously that, compared to mammals, *C. elegans* tends to have fewer genes with alternative transcripts and fewer alternative transcripts per gene [7]. This distinction is now emphasized with the interpretation that, even when multiple transcripts are expressed from a single *C. elegans* gene, these may all be expressed similarly and lack distinct functions in distinct cells, at least for transcription factor genes. In *Drosophila melanogaster* shadow enhancers have been identified, apparently to improve precision and reproducibility of gene expression patterns [28]. While these shadow enhancers are not envisaged to cause expression of alternative transcripts encoding distinct protein isoforms, the alternative promoters for *C. elegans* transcription factor genes could have a similar value for robustness of gene expression.

The low level of expression of transcription factor genes means that background noise makes up a larger fraction of the transcripts produced and increases the difficulty of distinguishing physiologically relevant transcripts for gene models. Simple detection of alternative transcripts, with the increasingly sensitive techniques available [29,30], need not mean that a transcript is functional or that production of alternative transcripts by a gene is physiologically important. This consideration applies to other organisms beyond *C. elegans*. Claims of functionality of alternative transcripts should depend on observation of phenotypes specifically dependent on the integrity of the proposed coding region of the alternative isoforms. Indeed reporter expression alone also does not reveal biological function for an alternative isoform. However, notwithstanding the difficulties arising from functional redundancy, complementation of gene knockouts with the subtly manipulated fosmids described here could address these questions of functional significance.

## Methods

### Recombineering

Fosmid clones were obtained from a *C. elegans* fosmid library (Source BioScience Life Sciences) of approximately 30-40 kb genomic DNA inserts cloned in the pCC1FOS vector. Fosmids, maintained at low copy number in EPI300 *E. coli*, were grown in overnight LB cultures containing 0.01% arabinose to induce clone copy number. DNA was extracted using either the FosmidMAX kit (Epicentre) or the QIAprep Spin Miniprep kit (Qiagen) and fosmid identity was confirmed by restriction digest.

Recombineering was carried out using a slightly modified protocol to that described previously [10]. Fosmid DNA was electroporated into either EL350 (Invitrogen) or MW005 [31] strains of *E. coli* and electrocompetent cells were prepared with incubation at 42°C to induce the Red functions. For each *gfp* insertion, a specific *rpsL-tetA(C)* cassette (RT cassette) was generated by PCR from a template containing the cassette flanked by the first and last 50 nucleotides of the GFP coding sequence (fUL#SB29 [11]) and maintained in the pCC1FOS vector without copy control induction. The primers consisted of 50 nucleotides of gene-specific sequence flanking the desired site of insertion, known as the homology arms (HAs), plus 20 nucleotides directed at the very 5′ or 3′ ends of the *gfp* present in the template. An extra nucleotide was inserted immediately before the initiation codon when the translational reading frame was to be shifted for assaying nested transcripts. For *unc-62*, substitutions were used to create in-frame stop codons in the alternative first exons that would knock out expression from transcripts containing the exon. To eliminate expression arising from transcripts containing the alternative internal exons in *unc-62*, a single base pair was inserted within that exon to create both an in frame stop codon and a translational frame shift. For introducing point mutations, the RT cassette was amplified in the same manner and with the same template except that: the primers consisted of 50 nucleotide HAs plus the first or last 20 nucleotides of the RT cassette sequence rather than the flanking GFP; and the HAs matched genomic DNA segments 200–300 base pairs apart, resulting in the replacement of this section by the RT cassette. PCR was performed with Platinum *Taq* DNA Polymerase High Fidelity (Invitrogen) and the products purified with a QIAquick Gel Extraction Kit (Qiagen). After electroporation and selection for RT cassette insertion, potential positive clones were re-streaked and screened directly by PCR with primers flanking the insertion point.

Single tetracycline-resistant RT-positive clones were used to prepare electrocompetent cells for the second recombineering step, again with Red functions induced. GFP coding sequence (with introns) was prepared by *Eco*RI digestion of a plasmid (pUL#SB94). Fragments of genomic DNA containing point mutations were amplified by PCR using the original purified fosmid as the template. The forward primer consisted of the same 50 nucleotide HA used to amplify the RT cassette with the desired mutation directly 3′ of the HA, followed by a further 18–22 nucleotides corresponding to the target. The reverse primer was a short 18–22 nucleotide oligonucleotide corresponding to the 5′ end of the reverse HA used to amplify the RT cassette. Cells were electroporated with the appropriate DNA, purified using a QIAquick Gel Extraction Kit (Qiagen), with selection for streptomycin resistance to detect the loss of the RT cassette. Potential positive clones were re-streaked and PCR screened. When using the EL350 strain, DNA was extracted and electroporated into the Copy Control strain EPI300. (MW005 contains both Red functions and Copy Control capability, and so transfer to EPI300 is then not necessary). Fosmid DNA was prepared and integrity confirmed by restriction enzyme digestion and/or sequencing. All primers were synthesized by IDT and long primers were PAGE-purified.

### Recombineering-mediated fosmid ‘stitching’

Recombineering was used to generate single fosmids, containing the whole of *daf-16* or *nurf-1*, from pairs of overlapping fosmids, each partially covering the gene, WRM0610bB12 and WRM065dE01 for *daf-16*, WRM0629dF11 and WRM0610dH04 for *nurf-1.* The recombineering technology was as described above but following the scheme presented in Additional file 4. An RT cassette was recombineered into the first fosmid named for each gene, within the region of overlap between the fosmids, for subsequent replacement with the second half of the gene from the second fosmid, in the second recombineering step. For *daf-16*, the inserts in both fosmids were in the same orientation with respect to the vector backbone, simplifying the manipulations. Due to the reverse orientation of the inserts for *nurf-1,* the RT cassette was generated by two PCR reactions to add in a 50 bp region corresponding to the end of the insert in the second fosmid. This then allowed the second half of *nurf-1* to be introduced into the first fosmid by recombineering. To address the reduced efficiency of recombineering with large fragments, a kanamycin cassette, amplified by PCR using primers with appropriate homology arms and pENTR201 (Invitrogen) as template, was inserted downstream of the target gene segments in the second fosmid, again by recombineering, selecting directly for kanamycin resistance. Fosmid DNAs containing the inserted kanamycin cassette were prepared and digested by restriction enzymes to release the target gene segments, *Sbf*I and *Afi*II for *daf-16* and *Sma*I for *nurf-1*. The gene segments were purified using the Qiaex II Gel Extraction Kit (Qiagen) for use in the second recombineering step. Replacement of the RT cassette with the downstream gene segment was selected for with kanamycin resistance. Potential clones were screened by PCR and the integrity of the resulting fosmids was confirmed by restriction enzyme digestion.

### *C. elegans* culture and transformation

*C. elegans* strains were maintained as previously described [32]. The wild type Bristol N2 strain [33] was transformed by microinjection [34] with fosmid DNA at 5–20 ng/ml and pRF4 plasmid DNA at 100 ng/ml. pRF4 contains *rol-6(su1006)*, conferring a rolling phenotype, allowing detection of transformants and maintenance of transgenic lines. The extrachromosomal arrays created in the transformation events typically contain both plasmid and fosmid DNA. Each transgenic line was derived from a different microinjected animal to ensure independence. GFP expression patterns were observed by epifluorescence microscopy with Chroma Technology Corp. filter set 41012 or Zeiss filter set 47 on a Zeiss axioplan microscope or on a Leica DMR microscope, respectively, equipped with DIC optics. Images were collected with a Photometrics CoolSNAP camera and Improvision Openlab software.

## Competing interests

The authors declare that they have no competing interests.

## Authors’ contributions

HC, JW and SB carried out the experimental work. CD and IAH conceived and coordinated the study. HC and IAH drafted the manuscript. All authors read and approved the final manuscript.

## Supplementary Material

Additional file 1 **Is a Table with the consideration of prior evidence for alternative transcripts for all potential *C. elegans *transcription factor genes, including: molecular and genetic gene names; mode of alternative transcript production; assessment of strength of evidence with comment; the class of transcription factor encoded; if there is no fosmid containing the gene available; and if the gene was selected for study.** Click here for file

Additional file 2 **Is a Figure containing the gene models for all *C. elegans *genes assayed including the exon/intron structure of each alternative transcript.** Click here for file

Additional file 3 Is a Table with details of all reporter gene fusions constructed and expression patterns obtained, including: molecular and genetic gene names; names of fosmid clones containing the reporter gene fusions; the name of one of the transgenic strains generated by transformation with each reporter gene fusion; precise details of the nature of the reporter gene fusions constructed; descriptions of the expression observed in the strains transgenic for each reporter gene fusion; and WormBase expression pattern identification number. Click here for file

Additional file 4 Is a Figure of the recombineering schema used to unite parts of large genes split across two fosmids into a large but single fosmid. Click here for file
