# Supplementary material for: The significance of alternative transcripts for Caenorhabditis elegans transcription factor genes, based on expression pattern analysis
Source: BMC Genomics. 2013 Apr 15;14:249. doi: 10.1186/1471-2164-14-249 (PMC3685541; doi:10.1186/1471-2164-14-249)

Additional Data File 2.

The gene models for all *C. elegans* genes assayed. The models are provided in alphabetical order by genetic gene name, apart from *F13H6.1* which does not have a genetic name yet and appears at the end. The gene models are as provided in WormBase WS190 with modifications made through to WS230 indicated. For each gene, the molecular gene names are included in brackets after the genetic gene name, with the additional final letter distinguishing the transcripts encoding distinct isoforms. The different colours of the exons indicate different orientations along the chromosomes. The scale bar in each panel is in base pairs along the respective chromosome.


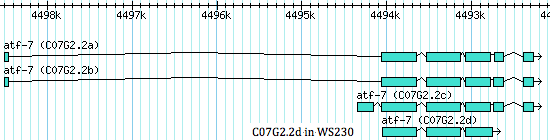


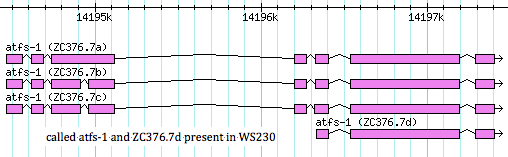


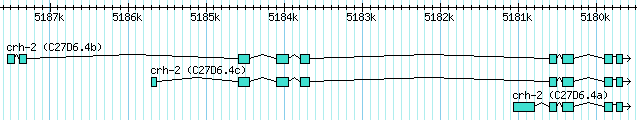


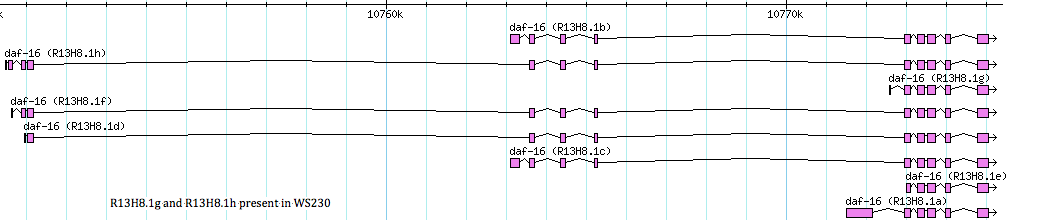


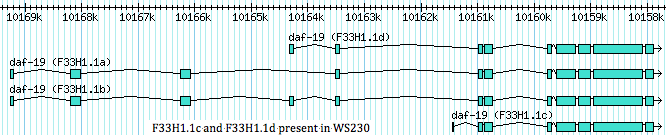


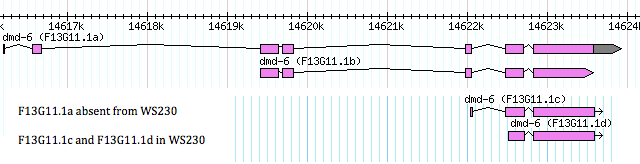


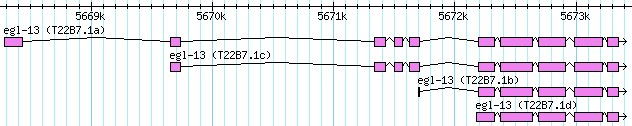


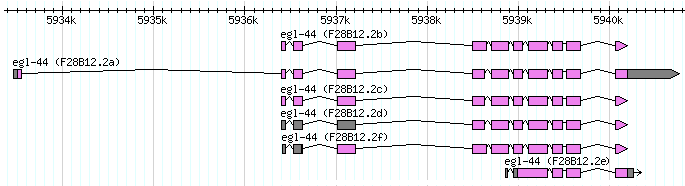


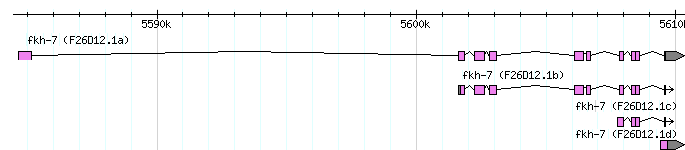


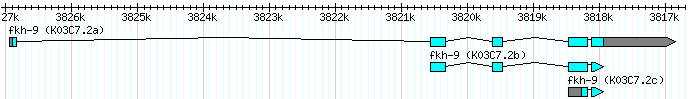


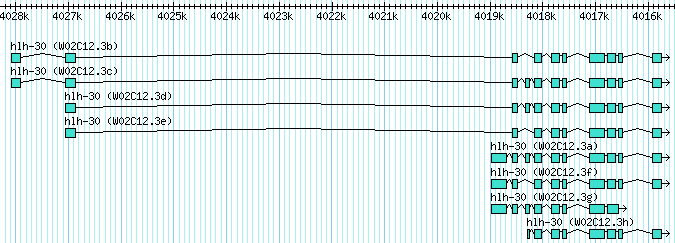


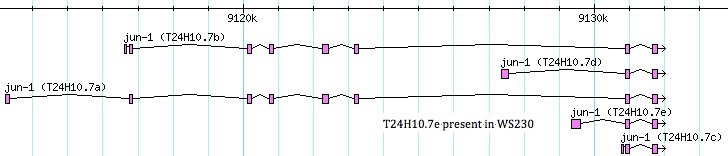


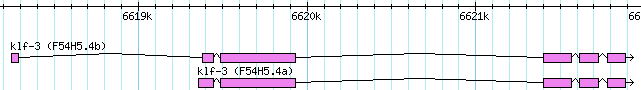


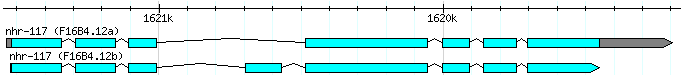


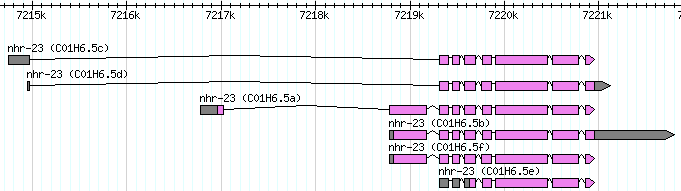


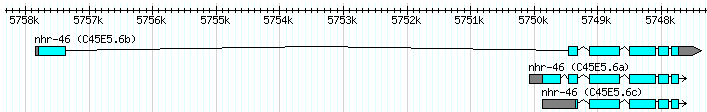


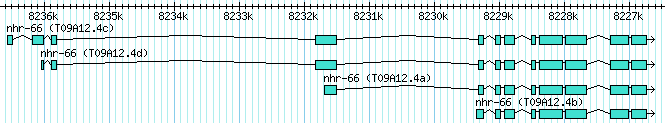


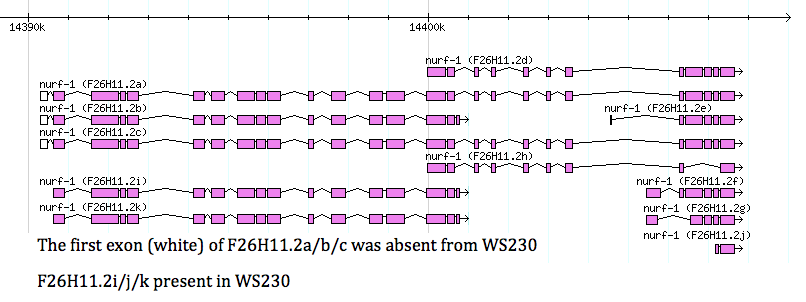


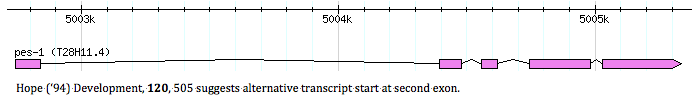


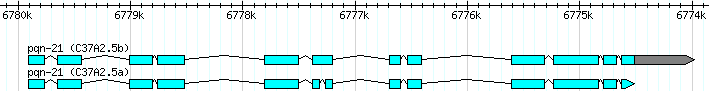


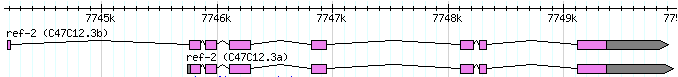


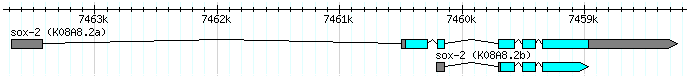


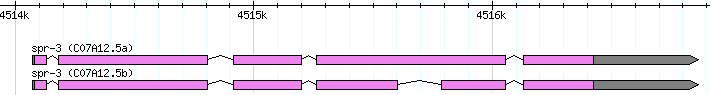


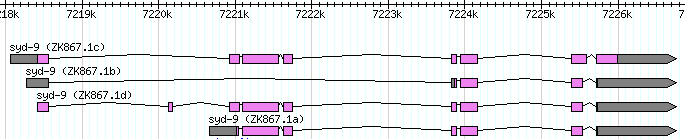


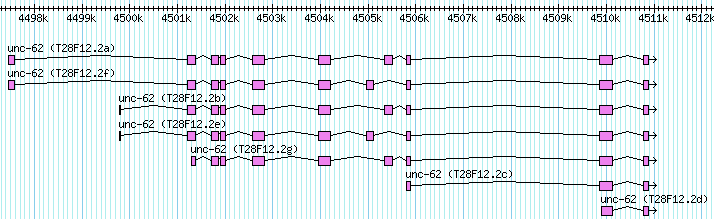


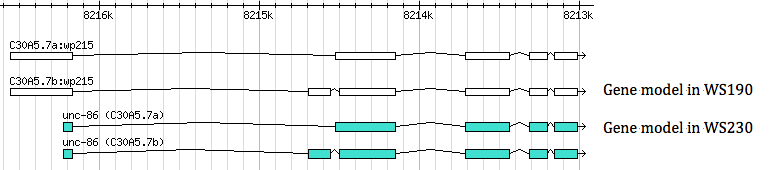


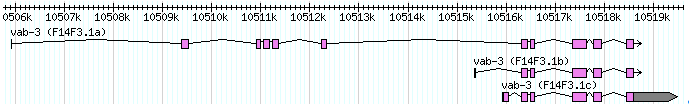


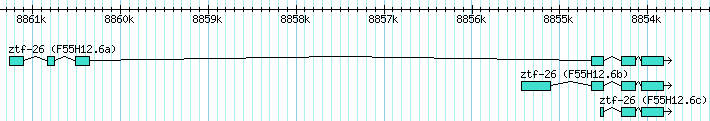


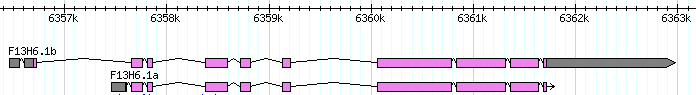

Supplement: Additional file 2 — Is a Figure containing the gene models for all C. elegans genes assayed including the exon/intron structure of each alternative transcript. [file 1471-2164-14-249-S2.docx]
