# Supplementary material for: The significance of alternative transcripts for Caenorhabditis elegans transcription factor genes, based on expression pattern analysis
Source: BMC Genomics. 2013 Apr 15;14:249. doi: 10.1186/1471-2164-14-249 (PMC3685541; doi:10.1186/1471-2164-14-249)

Additional Data File 4.

Recombineering schema to create intact copies of *daf-16* (A) and *nurf-1* (B) in single fosmids. The *rpsL-tetA(C)* (RT) and Kanamycin resistance (K^r^) selection cassettes were amplified by PCR before insertion by recombineering into the fosmids WRM0610bB12, WRM065dE01, WRM0629dF11 or WRM0610dH04 containing the *daf-16* 5’ end, the *daf-16* 3’ end, the *nurf-1* 5’ end or the *nurf-1* 3’ end respectively. The fosmids overlap (O) in the centre of each gene. Restriction enzyme digestion () generates the linear DNA fragments for the second step of recombineering to create fUL#HC131 for *daf-16* and fUL#HC88 for *nurf-1*. PCR primer sequences are provided below, 5'-3', with homology arms for recombineering in lower case, with complementarity for PCR priming in upper case, and with the gene / cassette / orientation (PCR 1 or 2) indicated.

Primer Sequence

daf-16/RT/For ttaagtctaacgcccggcgaccaatgcactgaaaacaacgttttgcccttTCGCTGTCGAGATATGACGGTG

daf-16/RT/Rev tgcaggtcgactctagaggatcccacaacgggcgaaaaaaggaggaaaaaGATGATAAGCTGTCAAACATGAG

daf-16/Kr/For atacagcgtcgcactgaaaaagagagtcatttcgcaaccttgcggcacggCCATCCAGCTGCAGCTCTGG

daf-16/Kr/Rev tgcaggtcgactctagaggatcccacaacgggcgaaaaaaggaggaaaaaTTAGAAAAACTCATCGAGCA

nurf-1/RT/For ggggaacaaaagcacagttaatacgaattcgacgccgtatccacaggctcTCGCTGTCGAGATATGACGGTG

nurf-1/RT/Rev(1) gggttagtaaaataacaacgtagtatataagatgaaatagtgagaaaataGATGATAAGCTGTCAAACATGAG

nurf-1/RT/Rev(2) atagaatactcaagcttgcatgcctgcaggtcgactctagaggatcccacGGGTTAGTAAAATAACAACG

nurf-1/Kr/For ataaccgttgattctctcgtagtctagtaaactttgaaataataggaaaaCCATCCAGCTGCAGCTCTGG


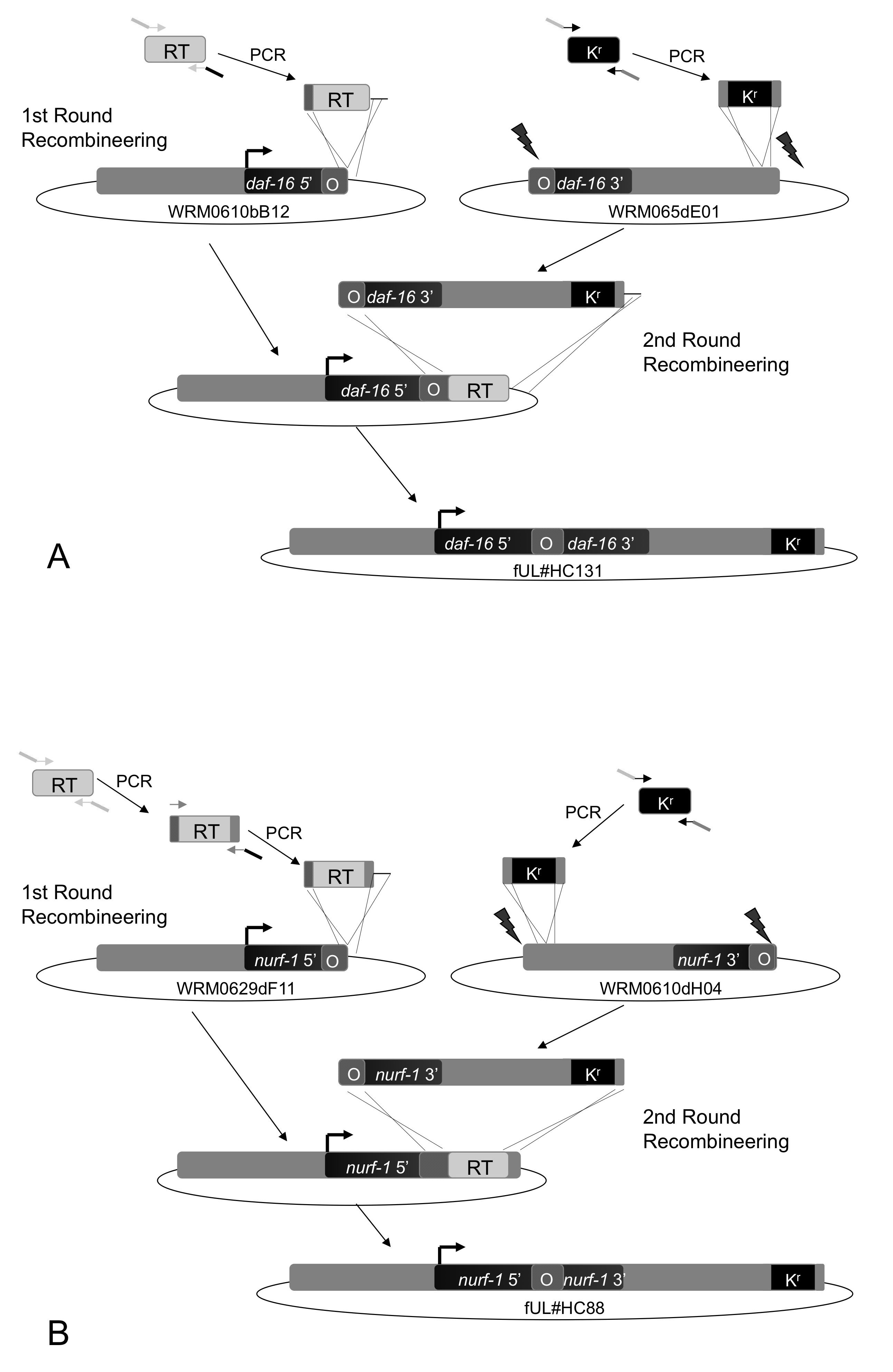

Supplement: Additional file 4 — Is a Figure of the recombineering schema used to unite parts of large genes split across two fosmids into a large but single fosmid. [file 1471-2164-14-249-S4.docx]
